# Lymphocytic Choriomeningitis Virus Infection in FVB Mouse Produces Hemorrhagic Disease

**DOI:** 10.1371/journal.ppat.1003073

**Published:** 2012-12-27

**Authors:** Frederick J. Schnell, Sarah Sundholm, Stacy Crumley, Patrick L. Iversen, Dan V. Mourich

**Affiliations:** 1 Sarepta Therapeutics, Corvallis, Oregon, United States of America; 2 Department of Microbiology, Oregon State University, Corvallis, Oregon, United States of America; Washington University School of Medicine, United States of America

## Abstract

The viral family *Arenaviridae* includes a number of viruses that can cause hemorrhagic fever in humans. Arenavirus infection often involves multiple organs and can lead to capillary instability, impaired hemostasis, and death. Preclinical testing for development of antiviral or therapeutics is in part hampered due to a lack of an immunologically well-defined rodent model that exhibits similar acute hemorrhagic illness or sequelae compared to the human disease. We have identified the FVB mouse strain, which succumbs to a hemorrhagic fever-like illness when infected with lymphocytic choriomeningitis virus (LCMV). FVB mice infected with LCMV demonstrate high mortality associated with thrombocytopenia, hepatocellular and splenic necrosis, and cutaneous hemorrhage. Investigation of inflammatory mediators revealed increased IFN-γ, IL-6 and IL-17, along with increased chemokine production, at early times after LCMV infection, which suggests that a viral-induced host immune response is the cause of the pathology. Depletion of T cells at time of infection prevented mortality in all treated animals. Antisense-targeted reduction of IL-17 cytokine responsiveness provided significant protection from hemorrhagic pathology. F1 mice derived from FVB×C57BL/6 mating exhibit disease signs and mortality concomitant with the FVB challenged mice, extending this model to more widely available immunological tools. This report offers a novel animal model for arenavirus research and pre-clinical therapeutic testing.

## Author Summary

Arenaviruses are carried by rodents, and in South America and West Africa can cause a fatal hemorrhagic fever syndrome in humans. Food, water or household items contaminated with rodent urine can be a source for transmission. General supportive care, anti-fever medication and the antiviral drug Ribaviran are used, however no treatment has proven effective. Due to the lack of small animal models capable of reproducing the human disease, development of an effective therapeutic has been slow. Here we report that a common laboratory arenavirus isolate, lymphocytic choriomeningitis virus, known to cause only a mild infection in humans and a chronic, wasting disease in most laboratory strains of mice produces a hemorrhagic-like disease in the FVB mouse strain. These mice exhibit signs of bleeding, multi–organ involvement, changes in blood diagnostics and mortality indicative of hemorrhagic fever syndrome following infection. We also show that a drug approach to reduce inflammation as a result of immune responses to the virus reduced disease signs and improved survival. Our study provides a small animal model for testing new treatment approaches and points to drug targets that lessen disease severity and improve survival from arenavirus induced hemorrhagic fever.

## Introduction

Viral hemorrhagic fevers (VHFs) are induced by viruses that belong to one of four families, *Arenaviridae, Bunyaviridae, Filoviridae, Flaviviridae.* The clinical symptoms of hemorrhagic fever vary depending on the severity and etiological agent but generally fever and bleeding are prominent manifestations of the disease. Hemorrhagic fever viruses, including arenaviruses, pose a significant public health threat both as emerging infectious diseases and as potential bioterrorism agents [1]. The majority of viruses in the *Arenaviridae* family require maximum biosafety containment (BSL-4) for handling which limits access for most researchers. In addition, the available animal models that induce hemorrhagic fever like symptoms require marmosets, hamsters, guinea pigs, primates, or immunocompromised mice [2], [3]. The lack of a non-immunocompromised mouse model for viral hemorrhagic fever makes it difficult to conduct pre-clinical drug screening. Mice are ideal for use in pre-clinical drug development because of their low cost and the extensive knowledge and reagents available for the species. There is a dire need for VHF therapeutic as there is no FDA approved drug available for hemorrhagic fever disease.

Ideally, the clinical course and signs produced in the animal model will parallel those observed in the human disease. The key characteristics of human viral hemorrhagic fever of arenaviral origin are multiorgan infections with hepatocellular necrosis and thrombocytopenia [2], [4], [5]. In this article, we report that the FVB strain of mice exhibits extreme susceptibility to hemorrhagic fever-like signs after LCMV-Clone 13 (LCMV-13) infection. FVB mice demonstrate thrombocytopenia, hepatocellular necrosis, petechiae, and death. This is in contrast to the C57BL/6 mouse strain's response to LCMV-13, which progresses to a chronic wasting disease [6]. FVB mice showed greatly increased (IL-6, IL-17 and IFN-γ) cytokine and (CXCL1 and MCP-1 and 3) chemokine production profiles early after infection compared to C57BL/6 mice and systemic TNF-α during the hemorrhagic phase of the disease. To investigate the underlying mechanism of the FVB pathology, separate groups of mice were either depleted of CD4+ or CD8+cells at time of infection. We found that mice deficient in either CD4+ or CD8+ cells maintained normal liver function and survived LCMV-13 infection. Furthermore, drawing from data produced in previous mouse HFV challenge experiments (e.g. Ebola or Marburg) we chose to examine the role of IL-17 responses in this arenavirus model. FVB mice treated to block IL-17 responses at the time of infection exhibited increased survival from LCMV-13 challenge compared to untreated mice. These data, to the best of our knowledge, describe the first mouse model of arenavirus induced hemorrhagic fever and support the possibility that T cell-mediated immunopathology plays a role in the underlying cause of HFV disease.

## Results

**Figure 1:**
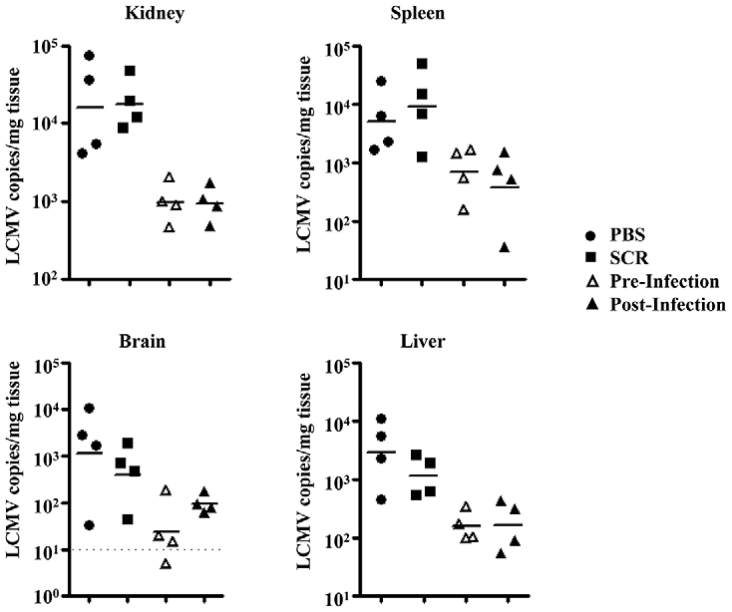
PMO*plus* targeting of LCMV in vivo inhibits viral replication. C57BL/6 mice were treated daily with 4 mg/kg antisense targeting the terminal sequences of LCMV starting either 2 days prior to infection (pre-infection) or 2 days post-infection or with a scrambled antisense sequence (SCR) 2 days prior to infection with 1–2×10^6^ p.f.u. LCMV clone-13. Viral load was quantified by qRT-PCR in tissue isolated from kidney, spleen, brain and liver 8 days post-infection. LCMV antisense PMO*plus* led to a significant reduction in viral RNA compared to PBS and scramble control in all tissues (Mann Whitney; p<0.05) except for pre-treatment compared to scramble in spleen and post-treatment compared to PBS in brain. Dotted line indicates lower level of assay detection. Solid lines indicate the mean LCMV copies/mg for each tissue type.

We initially sought to examine the anti-arenavirus activity of a modified PMO chemistry (PMO*plus*) that is complimentary to a sequence conserved within numerous arenavirus isolates, termed AVI-7012. The PMO*plus* chemistry has been shown to be efficacious in NHP filovirus lethal challenge models [7]. Conservation of the targeted sequence aligned to the L and S genomic segment and anti-genomic RNA is shown in Table 1. A similar targeting strategy demonstrated inhibition of arenavirus replication in vitro and in vivo utilizing a PMO conjugated to an arginine-rich peptide to enhance cellular uptake [8]. AVI-7012 (4 mg/kg) administered to C57BL/6 mice either prior to or following infection with LCMV-13 exhibited significant antiviral activity compared to PBS or scramble control treated mice when viral RNA was measured in kidney, spleen, brain and liver tissue (Figure 1). In pursuit of examining the uptake of PMO*plus* chemistry in vivo by cells that are known to support LCMV replication we employed a transgenic mouse model, which expresses enhanced green fluorescent protein (EGFP) as a positive readout of antisense activity via corrective splicing of the EGFP open reading frame [9]. As is common for many transgenic models this was produced on the FVB background strain of mouse.

**Table 1:** Sequence of AVI-7012 and alignment to Arenavirus L and S genome and anti-genome segments.

S Segment			
5′ terminus genomic RNA		5′ terminus antigenomic RNA	
AVI-7012	cgcgTggcAcctaggatccg	AVI-7012	cgcgTggcAcctaggatccg
SCR control	gcTcaggActccatgtggcc	SCR control	gcTcaggActccatgtggcc
Lassa	cgcgug***<$>\raster(73%)="rg1"<$>***caccuaggauccg	Lassa	cgcguggc***<$>\raster(73.5%)="rg2"<$>***ccuaggauccg
LCMV	cgcguggc***<$>\raster(73.5%)="rg2"<$>***ccuaggauccg	LCMV	cgcgug***<$>\raster(73%)="rg1"<$>***caccuaggauccg
Junin-XJ13	cgcgug***<$>\raster(73%)="rg1"<$>***caccuaggauccg	Junin-XJ13	cgcgug***<$>\raster(73%)="rg1"<$>***caccuaggauccg
Machupo	cgcgug***<$>\raster(73%)="rg1"<$>***caccuaggauccg	Machupo	cgcgug***<$>\raster(73%)="rg1"<$>***caccuaggauccg
Guanarito	cgcgug***<$>\raster(73%)="rg1"<$>***caccuaggauccg	Guanarito	cgcgug***<$>\raster(73%)="rg1"<$>***c***<$>\raster(73%)="rg1"<$>***ccuaggauccg
Sabia	cgcguggc***<$>\raster(73.5%)="rg2"<$>***ccuaggauccg	Sabia	cgcgug***<$>\raster(73%)="rg1"<$>***caccuaggauccg
WWA	cgcgug***<$>\raster(73%)="rg1"<$>***caccuaggauccg	WWA	cgcgug***<$>\raster(73%)="rg1"<$>***caccuaggauccg

**L Segment**			
5′ terminus genomic RNA		5′ terminus antigenomic RNA	
AVI-7012	cgcgTggcAcctaggatccg	AVI-7012	cgcgTggcAcctaggatccg
SCR control	gcTcaggActccatgtggcc	SCR control	gcTcaggActccatgtggcc
Lassa	cgcguggc***<$>\raster(73.5%)="rg2"<$>***ccuaggauccg	Lassa	cgcguggc***<$>\raster(73%)="rg1"<$>***ccuaggauccg
LCMV	cgcguggc***<$>\raster(73.5%)="rg2"<$>***ccuaggauccg	LCMV	cgcguggc***<$>\raster(73%)="rg1"<$>***ccuaggauccg
Junin-XJ13	cgcgug***<$>\raster(73%)="rg1"<$>***caccuaggauccg	Junin-XJ13	cgcgug***<$>\raster(73%)="rg1"<$>***caccuaggauccg
Machupo	cgcgug***<$>\raster(73%)="rg1"<$>***caccuaggauccg	Machupo	cgcgug***<$>\raster(73%)="rg1"<$>***caccuaggauccg
Guanarito	cgcgug***<$>\raster(73%)="rg1"<$>***caccuaggauccg	Guanarito	cgcgug***<$>\raster(73%)="rg1"<$>***caccuaggauccg
Sabia	cgcgug***<$>\raster(73%)="rg1"<$>***caccuaggauccg	Sabia	cgcgug***<$>\raster(73%)="rg1"<$>***caccuaggauccg
WWA	cgcguggc***<$>\raster(73%)="rg1"<$>***ccuaggauccg	WWA	cgcgug***<$>\raster(73%)="rg1"<$>***caccuaggauccg

Capitalized bases indicate positions where plus modifications have been introduced. Bold and italicized letters indicate positions where sequence mismatches are in viral target.

**Figure 2:**
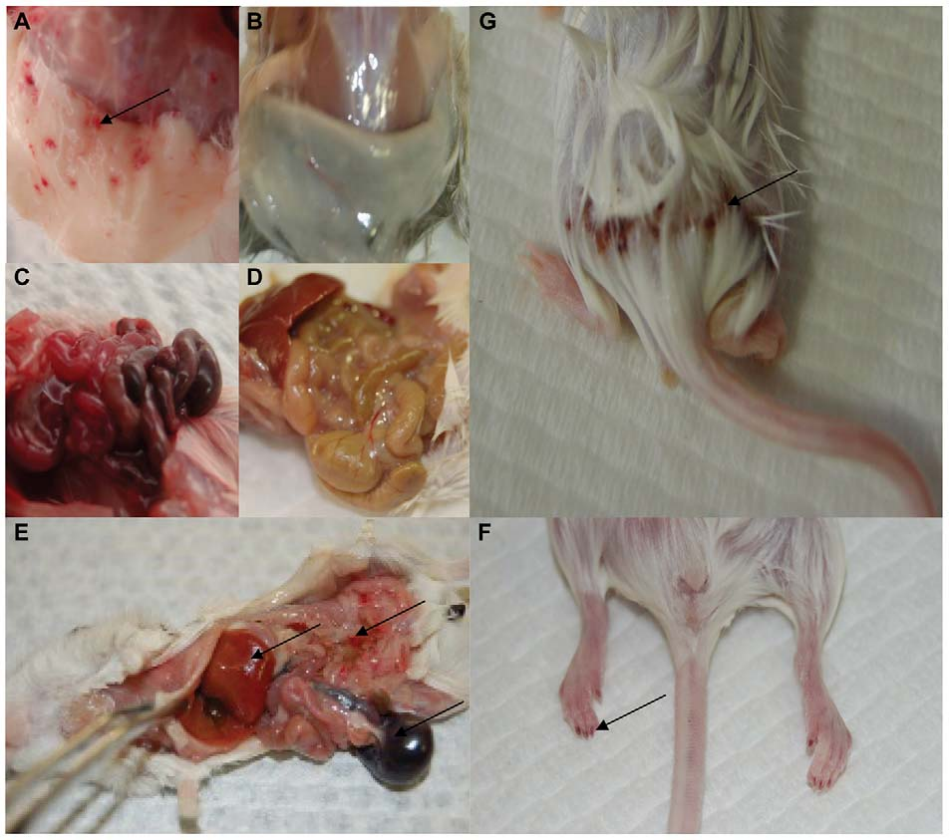
Overt signs of tissue morbidity and hemorrhage in the LCMV infected FVB mouse. Images were taken of FVB mice 6 to 8 days post-infection with LCMV -13. A) and B) Subcutaneous tissue in infected and uninfected FVB mice, respectively. C) and D) Gastro intestinal track of infected and normal FVB mice, respectively. E) General sites of blood pooling in the liver, stomach and GI track. F) and G) Cutaneous bleeding in the hindquarters and pooling of blood in the extremities, respectively. Arrows indicate sites of petechie and tissue hemorrhage.

**Figure 3:**
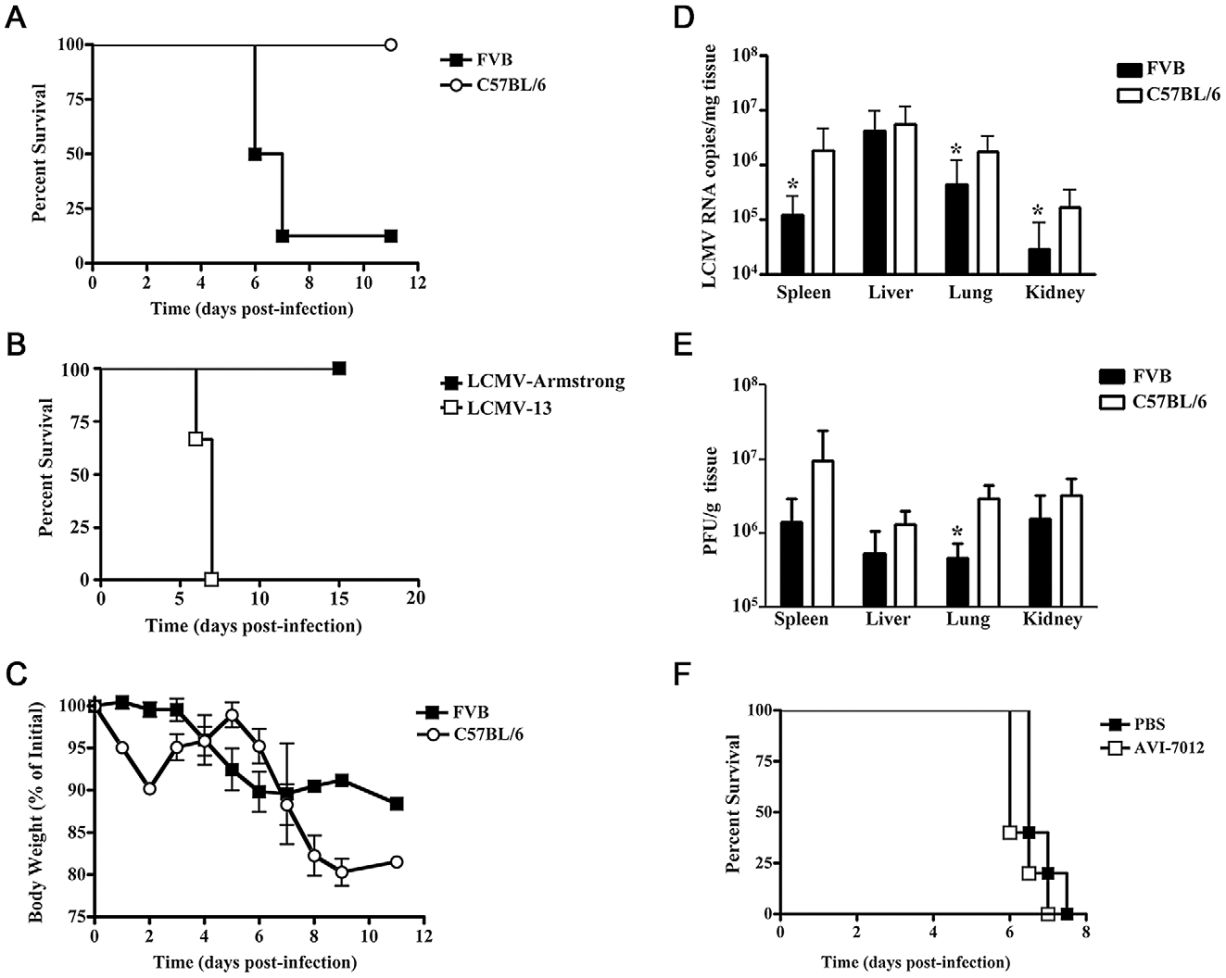
FVB mice develop an acute lethal disease after LCMV-13 infection independent of reduced viral titers in tissues. FVB or C57BL/6 mice were infected with 1–2×10^6^ p.f.u. and monitored for up to 11-days post-infection for development of disease signs or death. Animals were humanely killed upon development of severe disease. A) Survival curves of FVB and C57BL/6 mice (n = 11). B) Survival curves of FVB mice infected with LCMV-13 and LCMV-Arm (n = 3 mice) C) Weight change of FVB (n = 8) and C57BL/6 (n = 10) mice starting from pre-infection levels. Results are presented as a mean percentage of initial weight +/− standard deviation. D) Viral RNA in tissues of FVB (n = 13) and C57BL/6 (n = 15) mice infected with LCMV-13. Levels of viral RNA in indicated tissues at time of death were quantified by qRT-PCR for LCMV RNA. Results are presented as mean +/− standard deviation (*, p<0.05. One-way Anova). E) Viral burden as assessed by plaque assay. Infectious virus was quantified day 7 post-infection for all mice (n = 5). Results are presented as mean +/− standard deviation (*, p<0.05. t-test). F) FVB mice treated with anti-arenavirus antisense did not exhibit increased survival to LCMV infection compared to PBS treated animals (n = 5).

Four days post-infection, initial observations showed the infected EGFP mice react in an atypical manner compared to C57BL/6 mice at this point in a LCMV-13 infection. EGFP mice exhibited ruffled fur and showed signs of lethargy. Moreover, overt signs of severe disease were apparent 2–4 days later with some but not all of the infected EGFP animals presenting the following signs: mucosal, cutaneous and organ hemorrhaging (Figure 2) and decreased blood pressure, discordination, unresponsiveness, hypothermia, and seizures. The anomalous results observed following this LCMV-13 infection prompted us to carry out a second study without antisense treatment and with standard strains of C57BL/6 and FVB mice using the same virus stock in order to determine if the disease manifestation was due to the EGFP transgene. FVB mice displayed disease signs similar to those of the EGFP mice and on day 6 post-infection, death ensued and by day 8 post-infection only 1 out of 8 FVB mice infected with LCMV-13 had survived (Figure 3a). The FVB survivor did not clear virus, but instead harbored a long-term chronic infection with high viral load detected in multiple organs as late as day 36 post-infection. In agreement with previous reports, C57BL/6 mice showed 100% survival after LCMV-13 infection (Figure 3a). However, infection with high dose of the less pathogenic Armstrong strain of LCMV did not lead to any signs of hemorrhagic disease (Figure 3b). Weight loss in LCMV-13 infected FVB mice commenced after day 3 post-infection and FVB mice plateaued in weight loss at day 6 post-infection at 10.2% net weight loss (Figure 3c). The body weight pattern of C57BL/6 mice was strikingly different with a sharp drop in weight of 9.8% by day 2 post-infection, then a rebound in weight followed by a dramatic weight loss of 19.8% by day 9 post-infection (Figure 3c). FVB mice displayed a pantropic infection with virus being detected in multiple organs. However, even though FVB mice developed a moribund state while C57BL/6 mice did not, viral load was comparable or less in spleens, lungs, kidneys, and livers of FVB compared to C57BL/6 mice (Figure 3d–e). Yet C57BL/6 mice show no clinical symptoms of hemorrhagic-like disease, which indicates the pathology associated with LCMV infection in FVB mice is not solely virus mediated. We further examined the relationship of disease to viral load by targeting virus replication with antisense to Arenavirus 5′ termini, which has been shown to inhibit LCMV replication both in vivo and in vitro (Figure 1; [8]). Figure 1 shows that AVI-7012 can inhibit viral load in C57BL/6 mice, however, when FVB infected mice were treated to reduce viral load they did not exhibit a concomitant increase in survival (Figure 3f)

**Figure 4:**
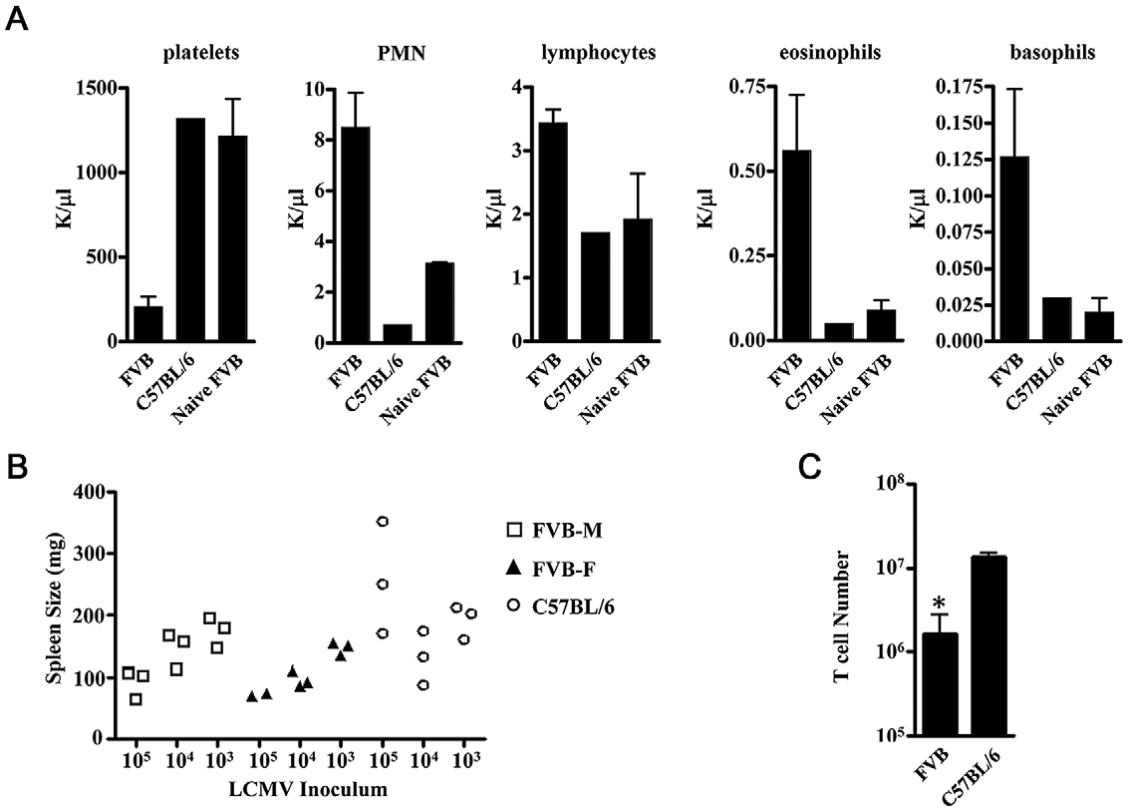
Effects of LCMV infection on blood and splenic cellularity in LCMV infected mice. A) Complete blood count with differential of LCMV-13 infected mice for platelet, neutrophil, lymphocyte, eosinphil and basophil counts. FVB (n = 4) or C57BL/6 (n = 1) mice were infected with 1–2×10^6^ p.f.u. LCMV-13 and blood was taken at day 7 post-infection. Additionally, naïve FVB (n = 2) blood was collected as a reference. Counts are presented as mean +/− standard deviation. Results for FVB mice are combined from two independent experiments. B) Spleen size in FVB mice exhibits an inverse correlation with infections dose of LCMV compared to C57BL/6. C) FVB mice have reduced T cell numbers as assessed by staining with anti-CD3 antibody in spleens after LCMV-13 infection. FVB (n = 10) or C57BL/6 (n = 8) mice were infected with 1–2×10^6^ p.f.u. LCMV-13 and spleens were harvested between day 7–11 post-infection. Results are presented as mean +/− standard deviation (*, p<0.05. t-test). Similar results were found in two additional independent experiments.

**Figure 5:**
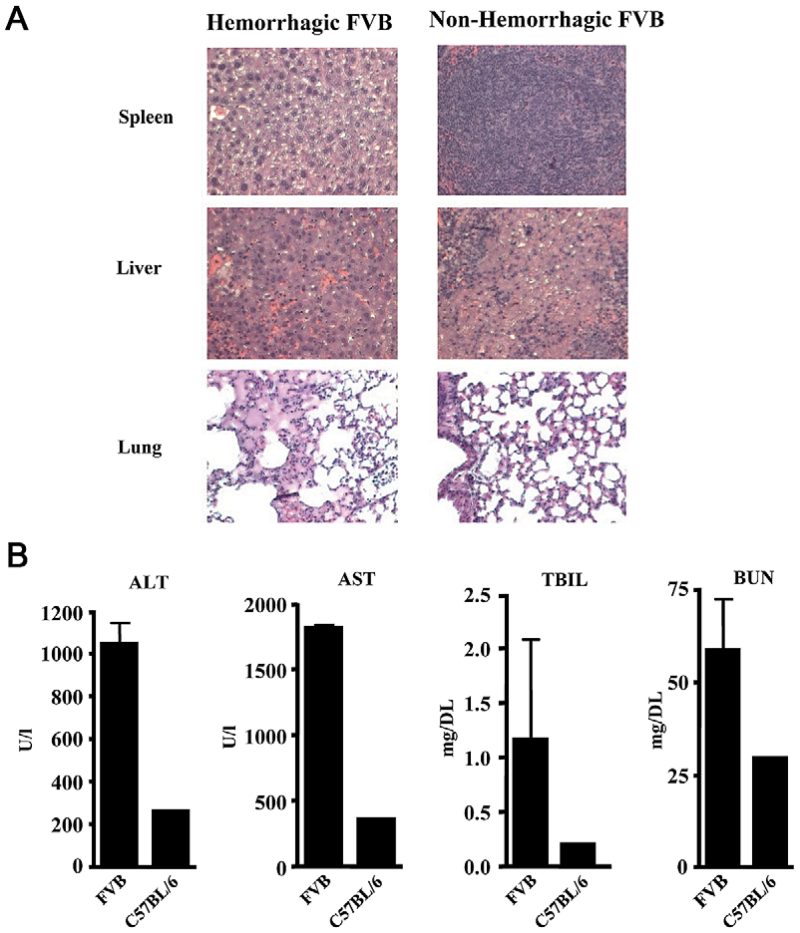
Indications of tissues damage in FVB mice following LCMV infection. FVB or C57BL/6 mice were infected with 1–2×10^6^ p.f.u. LCMV-13 and blood or tissue samples were taken at day 7 post-infection. A) Histological comparison of infected diseased and normal tissue in FVB mice. Signs of splenic, hepatic and pulmonary necrosis in FVB diseased mice in upper, middle and lower panels, respectively. B) Clinical chemistry profiles of alanine aminotransferase (ALT), aspartate aminotransferase (AST), total bilirubin (TBIL) and blood urea nitrogen (BUN) are shown. LCMV infected C57BL/6 data are presented as the values from combined serum collected from 4–5 mice. LCMV infected FVB data is presented as the mean +/− standard deviation of two groups of 3–5 mice. Results are representative of three independent experiments.

Considering the clear signs of hemorrhage in the FVB mice following infection we next sought to assess the hematologic parameters in FVB and C57BL/6 mice infected with LCMV-13. Similarly to the clinical manifestation to most arenaviral hemorrhagic diseases, platelet count differences were the most striking. FVB mice showed reduced platelet counts with a range of 146–324 K/ml compared to platelet counts of 1318 K/ml and 1216 K/ml for LCMV-13 infected C57BL/6 and naïve FVB, respectively (Figure 4a). Lymphocyte and granulocyte counts were dramatically increased in blood of LCMV-13 infected mice compared to naïve FVB and infected C57BL/6 mice (Figure 4a). However, spleen size was significantly smaller in LCMV-13 infected FVB mice, independent of gender, compared to infected C57BL/6 mice as well as total T cell counts in the spleen (Figure 4b and c). No mortality was observed in either gender at the challenge inoculum of 10^4^ p.f.u.. In accordance with the smaller spleens was the histopathological finding of severe splenic necrosis in LCMV-13 infected FVB mice while infected non-diseased FVB mice displayed no detectable necrosis (Figure 5a upper panels). Liver pathology in infected diseased mice revealed many single cells undergoing necrosis/apoptosis and randomly scattered zones of parenchymal necrosis with associated degenerate neutrophils (Figure 5a middle panels). Histological signs of modest alveolar edema and/or atelectasis were observed in the lungs of the infected FVB diseased mice but were absent in the infected non-diseased mice. Tissues from infected C57BL/6 mice showed no such disease indication except the livers of some C57BL/6 mice displayed rare tiny foci of hepatocellular degeneration and necrosis with scattered neutrophils (data not shown).

The following clinical biochemistry parameters were analyzed: alkaline phosphatase (ALK), alanine aminotransferase (ALT), aspartate aminotransferase (AST), calcium, cholesterol, triglycerides, albumin, creatinine, glucose, phosphorous, total bilirubin (TBIL), blood urea nitrogen (BUN), and total protein. LCMV-13 infected FVB mice had increased levels of ALT, AST, TBIL, and BUN compared to control mice indicating severe kidney dysfunction and hepatocyte destruction (Figure 5b). Calcium, cholesterol, triglycerides, albumin, creatinine, glucose, total protein, alkaline phosphatase, and phosphorous were normal in LCMV-13 infected FVB mice compared to controls (data not shown). These data combined demonstrate a pantropic infection leading to thrombocytopenia, cutaneous hemorrhaging, hepatic dysfunction, and ultimate death.

**Figure 6:**
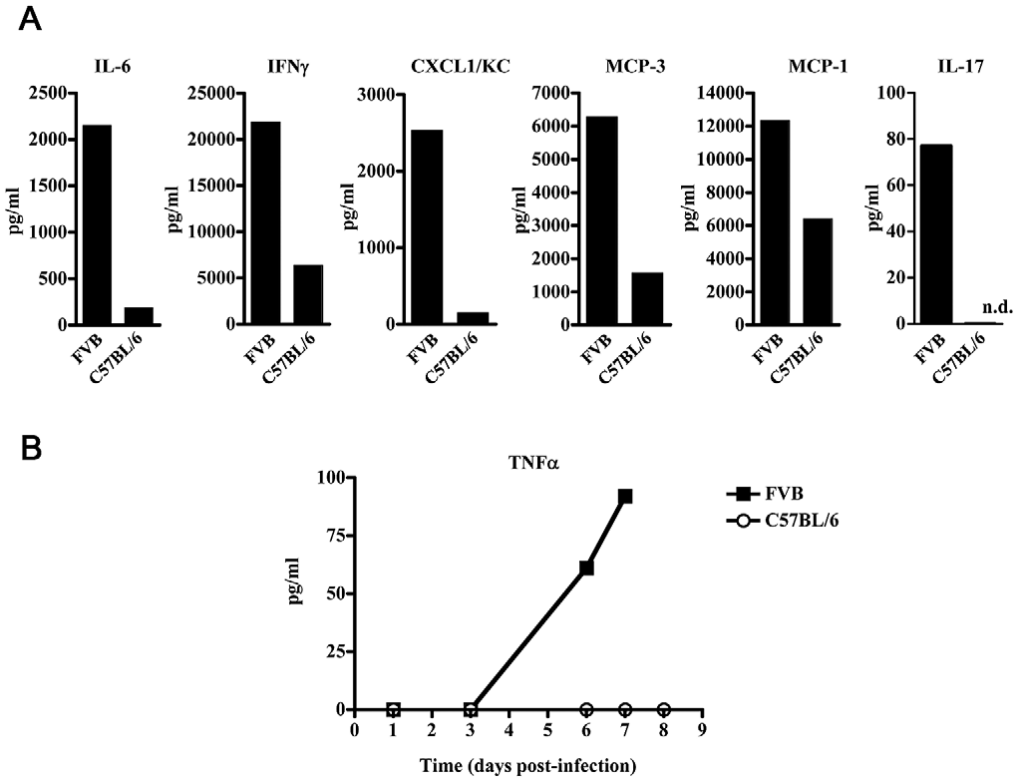
FVB mice infected with LCMV produce increased systemic cytokine and chemokine profiles. FVB or C57BL/6 mice were infected with 1–2×10^6^ p.f.u. LCMV-13 and serum harvested from 4–5 mice was combined and assayed for cytokine and chemokine levels. A) Serum levels of IL-6, IFN-γ, MCP-1, MCP-3 and CXCL1/KC measured 3 days post infection in FVB and C57BL/6 mice and IL-17 was measured day 1 post-infection. B) Serum levels of TNF-α were measured in FVB and C57BL/6 mice at indicated times post-infection. Results are representative of three independent experiments.

LCMV induces a well-characterized immunoregulatory state in most immunocompetent inbred mouse strains, including C57BL/6 mice [10] with subclinical disease signs. That FVB mice progress to hemorrhagic state and succumb implies a role of the immune response in the manifestation of the FVB hemorrhagic disease. To gain some insight into the inflammatory response prior to onset of hemorrhagic fever symptoms, we assayed for systemic cytokines in LCMV-13 infected FVB mice bled at day 3 post-infection and found increased levels of multiple pro-inflammatory cytokines and chemokines (Figure 6a). LCMV-13 infected FVB mice showed increased levels of IL-6 compared to C57BL/6 mice (2152 pg/ml versus 187 pg/ml), IFN-γ (2184 pg/ml versus 6399 pg/ml), CXCL1 (2530 pg/ml versus 154 pg/ml), MCP-1 (12342 pg/ml versus 6413 pg/ml), and MCP-3 (6284 pg/ml versus 1589 pg/ml). Strikingly, on day 1 post-infection FVB mice exhibited systemic IL-17A (78 pg/ml) while levels remained undetectable in C57BL/6 mice. At times during severe disease in FVB mice (day 6–8 post-infection), increased levels of systemic TNF-α were found compared to C57BL/6 mice (Figure 6b). TNF-α levels in LCMV-13 infected FVB mice were between 61–92 pg/ml whereas C57BL/6 mice had undetectable levels of TNF-α.

**Figure 7:**
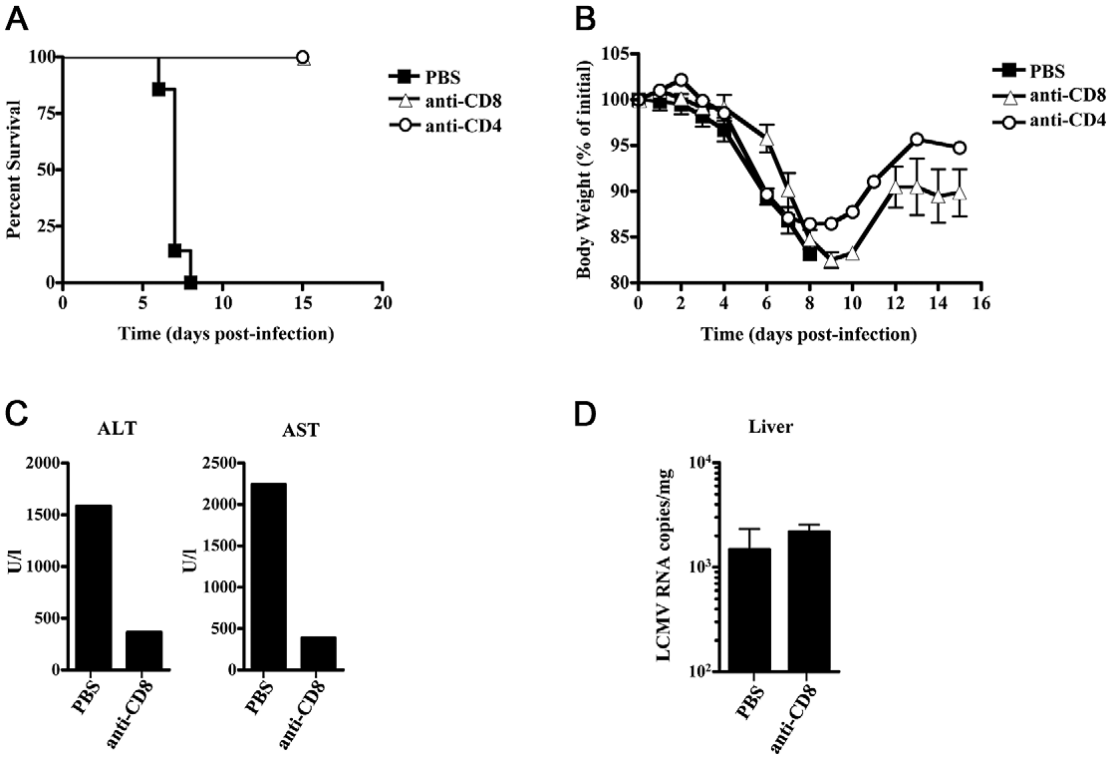
CD4 or CD8 depletion provides FVB mice protection from LCMV-13 induced death. FVB mice were treated with anti-CD8 antibody (n = 3), anti-CD4 antibody (n = 3) or PBS (n = 3) at days 0 and 1 post-infection with 1×10^6^ p.f.u. LCMV-13. Survival (A) and weight (B) were monitored daily until day 17 post-infection. Animals were humanely killed upon development of severe disease. C) Serum from 3 mice was combined and analyzed for AST and ALT levels. D) Viral burden in tissues measured after sacrificing animals. Levels of infectious virus in indicated tissue at time of death were quantitated by qRT-PCR for LCMV RNA. Results are presented as mean +/− standard deviation. Results are representative of two independent experiments.

In order to investigate the immune component of the FVB-related hemorrhagic disease further, we depleted either CD4+ or CD8+ cells in FVB mice with anti-CD8 or anti–CD4. Mice treated with anti-CD4 or anti-CD8 antibody at time of infection and 1 day after demonstrated 100% survival up to day 16 post-infection compared to 0% survival for PBS treated FVB mice (Figure 7a). While peak weight loss for anti-CD8 treated mice was similar to PBS treated mice (17.5+/−0.9% for anti-CD8 treated versus 14.6+/−0.2% for PBS treated), anti-CD8 treated mice regained weight after day 10 post-infection and plateaued at ∼10% lost body weight (Figure 7b). While peak weight loss was not as severe in anti-CD4 treated mice (13.5+/−5.8%), a similar trend was seen between anti-CD4 and anti-CD8 treated mice in that they began to regain weight around the same time PBS treated mice succumbed to disease. Anti-CD8 treated mice had lower AST (2245 u/ml vs 390 u/ml for PBS and anti-CD8 treated mice, respectively) and ALT (1584 u/ml vs 364 u/ml for PBS and anti-CD8 treated mice, respectively) readings than PBS treated mice indicating increased liver function (Figure 7c). Viral load in liver was similar between anti-CD8 treated and untreated mice (Figure 7d), which again suggests viral replication alone is not causing hemorrhagic disease.

**Figure 8:**
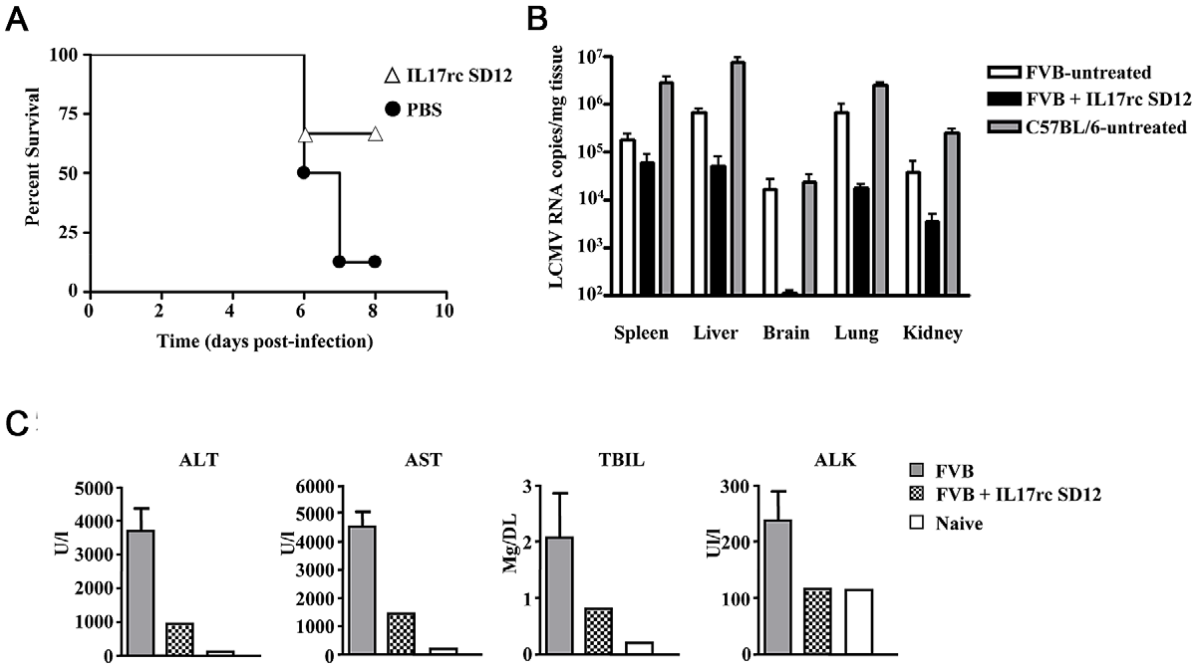
Blockade of the IL17 signaling pathway increases survival and reduces disease signs in LCMV infected FVB mice. FVB mice were infected with LCMV-13 and either treated with 7.5 mg/kg IL17RC SD12 antisense or PBS at days 0, 1, and 2 post-infection. A) Survival curves of PBS (n = 8) and IL17RC SD12 (n = 3) treated mice. B) Viral burden in tissues of FVB mice treated with PBS (n = 8) or IL17RC SD12 (n = 3). C) Clinical chemistry profiles of alanine aminotransferase (ALT), aspartate aminotransferase (AST), alkaline phosphatase (ALK), total bilirubin (TBIL) are presented. Untreated infected FVB data are presented as the mean +/− standard deviation of combined serum collected from three groups of 1–3 mice. Naïve FVB and IL17RC SD12 treated data are presented as the values from combined serum collected from 3–5 mice.

Taken together, hemorrhagic disease in this model appears to be caused by a skewed immune response. IL-17 has been shown to play a role in tissue destruction and we identified increased levels of systemic IL-17 early after infection (Figure 6). Previous data from our lab has shown antisense ablation of IL-17 receptor C (IL17RC) can prevent mortality in a mouse model of Ebola hemorrhagic disease. We, therefore, probed the role of IL-17 in arenavirus hemorrhagic disease. A delivery peptide conjugated antisense phosphorodiamidate morpholino oligomer (PPMO) was designed to target the splice-donor site of exon 12 of IL17RC (IL17RC SD12), thereby disrupting the translational reading frame of IL17RC, leading to reduced levels of surface IL17RC. Mice were treated with 7.5 mg/kg of IL17RC SD12 at day 0, day 1, and day 2 post-infection and monitored for disease symptoms. While only 12.5% of animals survived when treated with PBS, 66.7% of animals treated with IL17RC SD12 were still living at day 8 post-infection (Figure 8a). Viral load was significantly reduced in liver, lung, kidney, and brain with IL17RC SD12 treatment compared to untreated FVB (Figure 8b). Furthermore, IL17RC treatment protected liver and kidney function as seen by the reduced levels of ALT, AST, total bilirubin, and alkaline phosphatase found in serum (Figure 8c). Combined, these data suggest a role for IL-17 in mediating arenaviral hemorrhagic induced disease in FVB mice.

**Figure 9:**
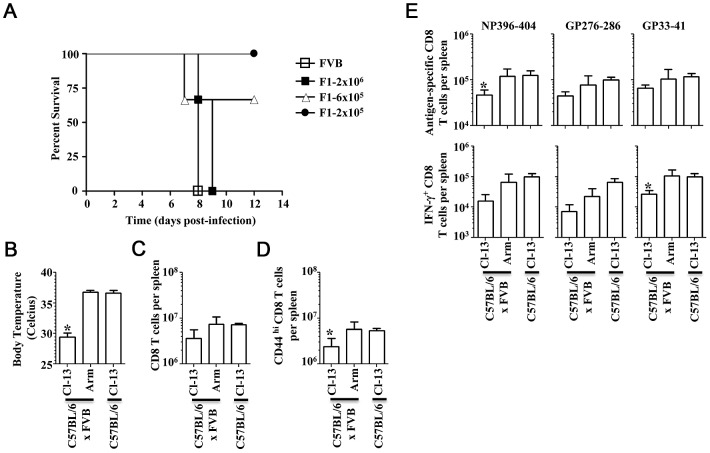
FVB×C57BL/6 F1 hybrids succumb to hemorrhagic disease while retaining H-2D^b^ restricted T cell response to LCMV infection. A) FVB mice were infected with 2×10^6^ PFU LCMV-13 and F1 hybrids were infected with the indicated doses of LCMV-13 and monitored for survival (n = 3). B) C57BL/6 or F1 hybrid mice were infected with the indicated virus and body temperature was assessed day 7 post-infection (n = 5). C–D) Splenic T cell numbers were assessed day 7 post-infection. E) Virus specific CD8 T cell numbers in the spleen as assessed by MHC pentamer stain (top) and IFN-γ production (bottom) after stimulation with the indicated peptides. Data are presented as the mean +/− standard deviation (*, p<0.05. t-test).

The FVB strain is useful for production of transgenic mice on inbred genetic backgrounds due to robust fecundity, and fertilized eggs contain large and prominant pronuclei facilitating microinjection of [11]. However, few immunological tools are available to probe the factors influencing hemorrhagic disease on the FVB H-2q background. We, therefore, bred C57BL/6 (H-2b) and FVB mice to create F1 hybrids. F1 hybrids were infected with a dose range of LCMV-13 and monitored for hemorrhagic disease symptoms. Mice infected with 2×10^6^ p.f.u. showed 100% mortality by day 9 post-infection, 66% mortality with 6×10^5^ p.f.u., and no mortality at 2×10^5^ p.f.u. (Figure 9a). We further confirmed hemorrhagic disease in F1 hybrids by infecting mice with either LCMV-13 or LCMV-Armstrong. Day 7 post-infection, C57BL/6 mice infected with LCMV-13 and F1 hybrids infected with LCMV-Armstrong demonstrated no clinical symptoms except for weight loss while F1 hybrids infected with LCMV-13 demonstrated a significant decrease in body temperature. F1 hybrids infected with LCMV-13 had a body temperature of 29.5+/−0.6 degrees Celsius while F1 hybrids infected with LCMV-Armstrong maintained normal body temperature of 36.8+/−0.4 degrees Celsius (Figure 9b). We then assessed LCMV-specific T cell responses in F1 hybrids. As can be seen in Figure 9d, F1 hybrids infected with LCMV-13 showed a significant reduction in CD44^hi^ CD8 T cell numbers (2.4+/−1.2×10^6^ cells versus 5.8+/−2.5×10^6^ cells for LCMV-13 versus Armstrong infected F1 hybrids, respectively). LCMV-13 infected F1 hybrids also demonstrated reduced LCMV-specific T cells as assessed by both MHC pentamer staining IFN-γ production (Figure 9e). F1 hybrids infected with LCMV-13 had significantly reduced numbers of Db/NP396–404 specific and Db/GP33–41 specific CD8 T cells as assessed by MHC pentamer staining and IFN-γ production, respectively (Figures 9e). These results confirm the findings from Figure 4 showing that mice undergoing hemorrhagic disease have lower T cell numbers late in infection.

## Discussion

One of the requirements for FDA approval of antiviral therapeutics is drug testing in accepted animal models that reproduce human disease as closely as possible. It has been thought that one of the fundamental features of LCMV biology is its ability to establish chronic infections in mice. This is in contrast to the disease course of LCMV in rhesus macaques, which succumb to hemorrhagic fever [12]. It has been reported that under certain circumstances LCMV can lead to mortality in mice. The New Zealand Black strain succumbs to a pulmonary disease much like hantavirus pulmonary syndrome [13]. Likewise, C57BL/6 mice infected with a medium dose of LCMV-13 displayed a similar lung pulmonary edema and interstitial mononuclear infiltration as NZB mice and 23% of those mice died [14]. Two other LCMV infection models have been reported to produce mortality with similar disease signs to the FVB model and a possible link to IL-17 production. The earliest was reported by Sarawar et. al, 1994. Here a subclinical infection of LCMV followed by low dose i.p. exposure to *Staphylococcus aureus* Enterotoxin B (SEB) resulted in a disease characteristic of hemorrhagic toxic shock leading to significant mortality in Vβ8.1 transgenic mice [15]. Although, Sarawar et. al would have not been able to measure IL-17 at the time, it has been shown in later studies that SEB will potently induce IL-17 expression in mice [16]. Additionally, large amounts of IFN-γ and IL-6, along with a transient increase in TNF-α were detected. Recently it has been shown the inflammatory effect of IL-17 on endothelial activation and neutrophil recruitment acts synergistically with TNF-α [17]. Both of these cytokines were produced in significant amounts in the FVB-LCMV hemorrhagic model and could account for the exaggerated inflammatory response in both models. Moreover, prior depletion of T cells gave similar results whereby the lethal effects of the LCMV infection with SEB were also greatly diminished. Although the source and precise role of IL-17 in the FVB-LCMV hemorrhagic model remains to be determined, anomalous production of IL-17 has been reported for mice deficient in T-bet and eomesodermin when infected with LCMV. These mice fail to differentiate LCMV-specific CD8+ killers T cells, required for defense against the virus, but instead produce a CD8+ IL-17-secreting lineage [18]. Upon viral infection, these mice develop a CD8+ T cell-dependent, progressive inflammatory and wasting syndrome characterized by multi-organ infiltration of neutrophils.

There is currently no mouse model that demonstrates multiple symptoms of the human disease of arenaviral hemorrhagic fever [2]. The disease of FVB mice infected with LCMV-13 described in this report mimics LCMV disease in macaques and many of the clinical signs of Argentine hemorrhagic fever (Table 2). However, the disease progression in FVB departs from the sequelae observed for Lassa Fever (Table 2). Viral load was not a good predictor of disease in our model while disease outcome is often predicted by viremia level in Lassa fever patients [19], [20], [21]. The smallest animal model currently used for arenaviral hemorrhagic fever is the guinea pig [2]. While this is a useful model in some respects, the major limitations of using guinea pigs for therapeutic testing and experimentation is the lack of information and reagents available for guinea pig analysis. Using the FVB inbred mouse strain opens up access to the plethora of tools available for mouse research. In addition, the cost and lower biosafety level (BSL-2+) using mice and LCMV-13, respectively, allows for high throughput screening.

**Table 2:** Clinical presentation of arenaviral hemorrhagic diseases in comparison to LCMV-13 infected FVB mice.

	AHV^a^	Lassa^b^	LCMV-WE^c^	LCMV-13/FVB
hepatocellular necrosis	+	+	+	+
elevated AST	+	+	+	+
hemorrhage	+/−	−	+	+
death	+	+	+	+
lymphopenia	+	+	+	+
thrombocytopenia	+	−	+	+
cytokine perturbation	+	+	+	+

a [32], [33].

b [20], [25].

c [12].

That FVB mice display an acute lethal disease after LCMV-13 infection while C57BL/6 mice develop a chronic infection reinforces the role of the host genetic factors in skewing the arenaviral disease course. Early in infection, we found increased levels of proinflammatory cytokines and chemokines in LCMV-13 infected FVB mice compared to C57BL/6 mice, which points to an immune component in the onset of hemorrhagic fever disease. It has been shown that LCMV-13 infected mice that were depleted of platelets develop lethal hemorrhagic anemia that is dependent on virus induced type I interferons [22] indicating a role for proinflammatory response in hemorrhagic disease. In addition, high levels of IL-6 and IFN-γ were found in rhesus macaques that succumb to lethal disease after LCMV infection [12]. One report has suggested that suppression of pro-inflammatory responses is partly responsible for the terminal shock associated with arenavirus infection in guinea pigs [23]. However, results from this study showed increased IFN-γ and MCP-1 in high pathogenic pichinde virus infected guinea pigs day 2 post-infection, indicating an early, robust proinflammatory response. Similarly, many of the proinflammatory cytokines and chemokines that were upregulated in LCMV-13 infected FVB mice early in infection were below C57BL/6 levels late in infection (data not shown).

That either CD4 or CD8 antibody treatment prevents death in all LCMV-13 infected FVB mice also supports an immune mediated component in the development of hemorrhagic disease. We believe that the disease is T cell mediated as waiting 3 days after infection before anti-CD8 treatment continued to protect mice from lethality (data not shown). Presumably LCMV has gone systemic by 3 days post-infection and the protective capability of anti-CD8 is T cell depletion rather than a CD8+ LCMV reservoir. The finding that CD4+ and CD8+ T cells are involved in the pathogenisis of LCMV-13 in FVB mice is in striking contrast to the C57BL/6 model. LCMV-13 induces an exhausted T cell phenotype where numerous inhibitory receptors are upregulated on CD8+ T cells, which lead to a chronic wasting disease [24]. However, LCMV-Armstrong infection in FVB mice mimics the disease progression of C57BL/6 with peak weight loss 8–9 days post-infection and then clearance of virus (Figures 3 and 9). This suggests that high viremia can skew the immune response. South American arenaviruses and Lassa induce splenic and lymphoid necrosis with varying degrees of lymphoid depletion [25]. While our T cell depletion studies point to a T cell-mediated pathogenesis in the FVB model, many signs in the FVB mice are consistent with human arenavirus immunosuppression such as splenic necrosis (Figure 5a), splenic involution (Figure 4b), and reduced T cell numbers (Figures 4c and 9). Our model suggests that skewing of the T cell response, possibly Th17, promotes unchecked inflammation, which then leads to splenic necrosis, lymphoid apoptosis, and lymphopenia. Our results are similar to Flatz et. al where mice that have humanized MHC class I develop severe Lassa fever whereas T cell depletion prevents disease [24]. We propose a three-tiered model similar to Flatz et. al where three outcomes are possible 1) potent T cell response controls virus (LCMV-Armstrong), 2) intermediate T cell response fails to control virus and triggers severe disease (FVB mice), 3) depletion of T cells allows persistence of Lassa virus with mild disease (T cell-depleted FVB mice).

The FVB mouse, named for its susceptibility to the B strain of Friend leukemia virus, exhibits a predisposition to several viral induced pathologies compared to other mouse laboratory strains [26]. Some examples are the neurological and immunological sequela observed subsequent to infection with either MOMuLV, a retrovirus, Theiler's a picornovirus and Minute virus, a parvovirus [27], [28], [29]. In contrast, the C57BL/6 (H-2Db) mouse strain is resistant to disease or persistence following infection with these viruses.

In the case of Theiler's virus disease, resistance has been linked to the MHC loci [30]. Specifically it has been shown that the FVB×C57BL/6 F1 and FVB H2-D^b^ transgenic mice are resistance to persistent Theiler virus infection and development of inflammatory lesions. This indicates that the H-2D^b^ allele confers dominance over H-2^q^. Our observation that the FVB×C57BL/6 F1 does not recapitulate resistance to LCMV infection or onset of hemorrhagic disease suggests that H-2D^b^ is not dominant in this condition of viral induced immunopathology. Although it is yet to be determined what immune related gene(s) influence the FVB and F1 mice susceptibility to hemorrhagic disease, a probative advantage prevails with the F1 hybrid. While maintaining the FVB-like hemorrhagic disease the F1 possesses an H2-D^b^ immune system, which will allow further dissection of the mechanism due to the availability of immunological reagents.

In summary, we have discovered a unique model for arenaviral hemorrhagic fever that could have broad applicability for arenaviral therapeutic development and arenavirus research. While our model does not mimic all hemorrhagic fever symptoms from viruses in the family *Arenaviridae*, the development of a hemorrhagic fever mouse model with an intact immune system represents a major advancement for arenavirus research and preclinical testing. In addition, our data suggests an immune mediated component to the onset of arenavirus hemorrhagic fever. If there is an immune component to the susceptibility to hemorrhagic fever, as our data suggests, the FVB and F1 hybrid LCMV-13 infection models will provide the tools necessary to decipher what the key factors are in initiating arenaviral hemorrhagic fever.

## Methods and Methods

### Ethics statement

Animal experiments were conducted to comply with the Public Health Service (PHS) *Policy on the Humane Care and Use of Laboratory Animals*, the US Department of Agriculture's (USDA) Animal Welfare Act & Regulations (9CFR Chapter 1, 2.31), the Animal Care and Use Review Office (ACURO), a component of the US Army Medical Department and Medical Research and Material Command USAMRMC and the United States Government Principles for the Utilization and Care of Vertebrate Animals Used in Research, Teaching and Testing with prior approvals for established protocols from the Oregon State University Institutional Animal Care and Use Committee and ACURO when appropriate.

### Mice and LCMV infections

C57BL/6 and FVB mice were purchased from Jackson Laboratories. C57BL/6 and FVB mice were bred to create F1 hybrids at Oregon State University Department of Animal Resources. All mice were housed at Oregon State University Department of Laboratory Animal Resources facility and experiments were conducted according to approved Institutional Animal Care and Use Committee protocols. Mice were used at 5–8 wks of age. Mice were infected with 1–2×10^6^ p.f.u. LCMV-clone 13 or LCMV-Armstrong. Standardized recording of death and disease symptoms was performed on a daily basis. Symptoms of severe disease were hunched posture for more than 24 hours without movement, discordination, and shaking upon movement. For CD8 depletion, mice were treated with 0.5 mg clone 53–6.7 anti-CD8 antibody at time of infection and 24 hours post-infection. For anti-CD4 depletion, mice were treated with 0.3 mg CD4 antibody clone GK1.5. For IL17RC SD12 antisense treatments, mice were dosed with 7.5 mg/kg intraperitoneal route on days 0, 1, and 2. IL17RC SD12 sequence is CTG GAC ACA GAG GTT GG. The PMO*plus* and PPMO compounds used were manufactured as previously described, respectively [7], [8].

### Viral quantification

Tissues were weighed and 300 ml of DMEM was added per tube. A stainless steal bead was added and tissues were homogenized in a Tissue Lyser (Qiagen) for 3 min at 20 Hz. Kidneys were digested in DMEM+1 mg/ml collagenase for 30 min prior to homogenization. After homogenization, tissues were spun down at 14 k for 5 min and 50 µl sup was taken for RNA isolation with Magmax Blood RNA Isolation Kit (Ambion) according to manufacturer's instructions. Viral load of the spleen, kidney, liver,and lung was determined by qRT-PCR amplification of viral GPC [forward primer (5′-GCAAAGACCGGCGAAACTAG-3′), reverse primer (5′-CGGCTTCCTGTTCGATTTGGT-3′) and a taqman probe (5′-CCCAAGTGCTGGCTTGTCACCAAT-3′)]. To translate the qRT-PCR results from a cycle threshold (CT) value into copy number, a standard curve was generated using PCR product of the GP amplicon. The GP amplicon PCR product was run on a gel, excised and purified, then spectrophotometry was used to quantify a copy number for that CT value. Plaque assays were conducted as previously described [31].

### CBC and clinical chemistry analysis

Blood was collected through retroorbital puncture using capillary tubes on anesthetized mice directly before sacrifice. Serum was collected after spinning at 8 k for 5 min. Blood and serum were sent to Charles River Laboratories for CBC with differential and Clinical Chemistry profiles.

### Cytokine analysis

Serum was assayed for cytokine and chemokines using Flow Cytomix bead system (Bender Medsystem) according to manufacturer's protocol.

### Pentamer ICS analysis

Db/GP33, Db/GP276, and Db/NP396 pentamers were purchased from Proimmune (Sarasota, FL). Single cell suspensions were stained with pentamer according to manufacturers protocol and then subsequently stained for CD8, CD44, and CD19 antibodies (BD Biosciences, San Jose, CA). Pentamer positive cells were detected on FC500 (Beckman Coulter, Indianapolis, ID) and analyzed on FlowJo software (Ashland, OR). For ICS analysis, splenocytes were stimulated overnight with 1 µg/ml of the indicated peptides. BFA (Ebioscience) was added 4 hours prior to harvest and cells surface stained for CD44 and CD8, fixed in cytofix/perm and washed in perm/wash buffer (BD Biosciences). Cells were then incubated with IFNγ antibody (BD Biosciences).
